# Protein disulfide isomerase a4 promotes lung cancer development via the Stat3 pathway in stromal cells

**DOI:** 10.1002/ctm2.606

**Published:** 2022-02-15

**Authors:** Tzung‐Yan Chen, Chun‐Yen Yang, Meng‐Ting Yang, Tien‐Fen Kuo, Cicero Lee‐Tian Chang, Chih‐Li Chen, Tsung‐Han Lee, Greta Yang, Wen‐Chin Yang, Ching‐Feng Chiu, Alex Yang‐Hao Yu

**Affiliations:** ^1^ Agricultural Biotechnology Research Center Academia Sinica Taipei Taiwan; ^2^ Institute of Biotechnology National Taiwan University Taipei Taiwan; ^3^ Department of Life Sciences National Chung Hsing University Taichung Taiwan; ^4^ Department of Veterinary Medicine National Chung‐Hsing University Taichung City Taiwan; ^5^ Institute of Pharmacology National Yang‐Ming University Taipei Taiwan; ^6^ Graduate Institute of Integrated Medicine China Medical University Taichung Taiwan; ^7^ Graduate Institute of Metabolism and Obesity Sciences College of Nutrition Taipei Medical University Taipei Taiwan; ^8^ Division of Pulmonology, Chang‐Hua Hospital Ministry of Health and Welfare Changhua Taiwan

**Keywords:** cancer stroma, lung cancer, Pdia4, Stat3

## Abstract

**Background:**

Protein disulfide isomerases a4 (Pdia4) is known to be involved in cancer development. Our previous publication showed that Pdia4 positively promotes cancer development via its inhibition of procaspase‐dependent apoptosis in cancer cells. However, nothing is known about its role in the cancer microenvironment.

**Results:**

Here, we first found that Pdia4 expression in lung cancer was negatively correlated with patient survival. Next, we investigated the impact of host Pdia4 in stromal cells during cancer development. We showed that Pdia4 was expressed at a low level in stromal cells, and this expression was up‐regulated akin to its expression in cancer cells. This up‐regulation was stimulated by tumour cell‐derived stimuli. Genetics studies in tumour‐bearing wild‐type and *Pdia4^–/–^* mice showed that host Pdia4 promoted lung cancer development in the mice via cancer stroma. This promotion was abolished in *Rag1^–/–^* mice which lacked T and B cells. This promotion could be restored once T and B cells were added back to *Rag1^–/–^* mice. In addition, host Pdia4 positively regulated the number and immunosuppressive function of stromal cells. Mechanistic studies showed that host Pdia4 positively controlled the Stat3/Vegf pathway in T and B lymphocytes via its stabilization of activated Stat3 in a Thioredoxin‐like domain (CGHC)‐dependent manner.

**Conclusions:**

These findings identify Pdia4 as a possible target for intervention in cancer stroma, suggesting that targeting Pdia4 in cancer stroma is a promising anti‐cancer approach.

## Graphical abstract

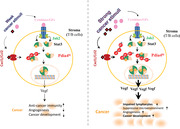

## 1 INTRODUCTION

Cancer is a leading worldwide cause of death. In 2020, 14 million new cases and 10 million cancer deaths were reported worldwide.^1^, ^2^ Cancer is composed of cancer cells and host stromal cells. Host stromal cells in the tumour microenvironment play a pivotal role in tumour initiation, progression and metastasis.^3^ Stromal cells are a heterogeneous cell population that includes cancer‐associated fibroblasts, endothelial cells, leukocytes, etc. The progression of tumours and their metastasis which is partly caused by the crosstalk between cancer cells and the stroma has been well‐studied.^4^ An understanding of the host stroma contribution to cancer development will broaden our knowledge about the related signaling pathways. Along with cancer‐targeted therapies, stroma‐targeting therapy is also an option for cancer therapy because of its genetic stability and universal application.^5^ However, therapeutic targets available for cancer stroma are currently limited.

Accumulating evidence shows that cancer cells educate the infiltrating leukocytes in tumour sites to support cancer growth and survival.^6^ For instance, tumour‐infiltrating dendritic cells, regulatory T cells, and myeloid‐derived suppressor cells are involved in immunosuppressive activities. In contrast, other immune cells are known to be functionally impaired by cancer‐associated antigens such as antigen‐specific CD8^+^, CD4^+^ helper T or B lymphocytes.^7^ Soluble mediators from cancer cells and stromal cells such as Cxcl1, Ccl2, Vegf family, Tgf‐β and GM‐CSF are reported to participate in tumour development.^8^ Vegfa, Vegfb and Vegfc are encoded from different *Vegf* genes.^9^ Vegfa, Vegfb and Vegfc are ligands for Vegfr1, Vegfr2 and Vegfr3.^10^, ^11^ Vegfa and Vegfc have been reported to suppress the function of T cells.^12^, ^13^ In addition, some transcription factors like signal transducer and activator of transcription 3 (Stat3) were initially identified as oncogenes in tumour cells.^8^ Stat3 is activated in cancer and stromal cells by inflammatory stimuli. The regulators of oncogenes are potential therapeutic targets for cancer.

**HIGHLIGHTS**

1. Expression of protein disulfide isomerases a4 (Pdia4) in cancer stroma is up‐regulated by cancer stimuli (e.g., Cxcl1 and Ccl2).
2. Pdia4 promotes lung cancer development via regulation of the Stat3/Vegf axis in T and B lymphocytes of cancer stroma.
3. This regulation involves stabilization of phosphorylated Stat3 by interaction with Pdia4 in the Pdia4/Stat3/Vegf cascade.

Given the importance of protein homeostasis, chaperones are emerging as important players in health and diseases in humans. The protein disulfide isomerases (Pdis) are a group of chaperones with multiple functions.^14^ The majority of them have an Endoplasmic reticulum (ER) retention sequence at their C terminals.^14^, ^15^ However, accumulating data showed that apart from the cytosol (ER and other organelles), Pdis reside in the nucleus and membrane of different cell types and plasma.^16^, ^17^ Thus, Pdis are thought to possess ER‐relevant and ‐irrelevant localizations and function like other ER chaperones.^18^ They are implicated in cell viability/growth, infection, fertilization, coagulation, immunity and cancer.^18^ The role of Pdis in cell growth is poorly studied, and the mechanism by which Pdis regulate cell growth is still elusive. Genetics studies in yeasts demonstrated that Pdi1 is essential for cell viability. However, Pdi1 and the other 4 Pdis may not be functionally interchangeable in yeasts.^16^ Of note, mammalians have 21 Pdi members with oxidoreductase, disulfide isomerase and chaperone activities. Among them, Pdia4 is structurally the largest Pdi with three CGHC motifs.^14^, ^16^ Unlike Pdia1, Pdia4 is not an essential gene because its knockout mice had no noticeable phenotypes.^16^ We and other groups showed that it suppresses cell death in cancer cells.^16^, ^19^ However, the role of Pdia4 in stromal cells (i.e., host Pdia4) but not cancer cells during cancer development is completely unknown.

Here, we first investigated the expression of host Pdia4 in the cancer stroma and its expression up‐regulation during lung cancer development. Next, we characterized the role of host Pdia4 in cancer development using lung cancer‐bearing wild‐type (WT) and *Pdia4^–/–^* mice on the B6 and *Rag1^–/–^* backgrounds. The molecular basis of cancer stroma‐related Pdia4 for cancer development was elucidated by microarray analysis, ingenuity pathway analysis (IPA), real‐time polymerase chain reaction (RT‐PCR) analysis, immunoblotting analysis, flow cytometry, domain mapping, and dual luciferase reporter assays.

## 2 MATERIALS AND METHODS

### 2.1 Cells, reagents and plasmids

GK1, a Green fluorescent protein (GFP)‐expressing Lewis lung carcinoma cell line, Jurkat cells (CRL‐2899), a human leukemia T cell line, and Raji cells (CRL‐7936), a human B lymphoma cell line were grown in Roswell Park Memorial Institute 1640 medium (RPMI) medium with 10% bovine serum. Stromal cells were isolated from mouse stromata or tumour sites of GK1 tumour‐bearing mice. Protamine sulfate, puromycin dihydrochloride, β‐mercaptoethanol, dual luciferase assay kit and trypsin were purchased from Promega (WI, USA). Lipopolysaccharide (LPS), collagenase and DNase were purchased from Sigma (NY, USA). Vegf, Cxcl1 and Ccl2 proteins were purchased from R&D Systems (MN, USA). His‐Stat3, a recombinant protein containing a His‐tagged Sta3, was purchased from Abcam (UK). His‐tagged phospho‐Stat3 was prepared by transfecting pHis‐Stat3, an expression plasmid containing a His‐tagged Stat3 cDNA, into Jurkat cells using TransIT‐X2 (Mirus Bio, WI, US). After 24 h, the cells were treated with 50 μM pervanadate for 30 min. His‐tagged phospho‐Stat3 was isolated from the cells and checked for its phosphorylation. Antibodies are listed in Table S1. Tumour conditioned medium was prepared from culture medium of GK1 cells (5 × 10^6^) that were grown in 20 ml RPMI containing 10% bovine serum for 24 h, and the medium was filtered using 0.22 μ filters and aliquoted for long‐term storage at −80°C freezers. His‐Pdia4 (Enzo, NY, USA) and His‐VP3 composed of His‐tagged viral protein 3 of foot mouth disease virus were as described previously.^16^ Gst‐Pdia4 and Gst‐Pdia4^280‐646^ were constructed by cloning full‐length Pdia4 cDNA and cDNA of N‐terminal‐truncated cDNA of Pdia4, corresponding to aa 280 to 646, to pGEX4T3, and the Gst funstion proteins were produced according to the manufacturer's manual (GE Healthcare, IL, USA). Constructs expressing Flag‐tagged Pdia4 and its mutants as well as pβ‐Actin‐RLuc have been described elsewhere.^16^ Plasmids, pENTER‐Stat3 and pStat3Y705F‐TAL‐FLuc, were purchased from ViGene Biosciences (MD, USA) and Addgene (MA, USA), respectively. The plasmid, pStat3‐3×BS‐TAL‐FLuc, was constructed by an insertion of a trimeric Stat3 binding site to pStat3Y705F‐TAL‐FLuc, followed by a point mutation from phenylalanine to tyrosine at 705. The plasmid, p3×SBS‐TAL‐FLuc, was derived from pStat3‐3×SBS‐TAL‐FLuc. His‐tagged Stat3 (pHis‐Stat3) and its deletion mutants were constructed by an insertion of full‐length or partial cDNA of Stat3 into p12×His‐cDNA3.

### 2.2 Immunohistochemical staining of lung cancer tissues of human and mouse origin

Lung tissues of healthy volunteers and patients with lung cancer were purchased from Biomax (T045e). The human specimens were stained with hematoxlin and eosin (H&E) and visualized using a light microscope. Healthy lung and stroma‐rich versus stroma‐poor cancer lung were differentiated based on H&E staining. The level of Pdia4 in health lung and lung cancer was quantified with Pdia4 signal of the whole spot of lung cancer. Pdia4 scores were calculated based on four grades: 0, tissues without Pdia4 signal; 1, 10% or less tissues had Pdia4 signal, 2, 10%–25% of tissues had Pdia4 signal; and 3, >25% of tissues had Pdia4 signal. Furthermore, the same specimens were stained with antibodies against Pdia4 and CD45 and 4′,6‐Diamidine‐2′‐phenylindole dihydrochloride (DAPI) and visualized using a fluorescent microscope. Alternatively, mouse tumour tissues were fixed with formalin, embedded with paraffin and stained with the indicated antibodies. The slides were developed by 3, 3′diaminobenzidine tetrahydrochloride (DAB) and counterstained with hematoxylin, photographed and analysed using the Axio Vision program (Carl Zeiss, Germany). The number of cells per microscopic field was counted based on H&E signal. The percentage of antibody signal per microscopic field was obtained based on the formula, % = 100% × pixels of the indicated antibody divided by pixels of the microscopic field.

### 2.3 Animal studies

All mice had free access to chow and water and were housed at 21–23°C with 12 h light‐12 h dark cycles. All mice were handled in accordance with the guidelines laid out by the Academia Sinica Institutional Animal Care and Utilization Committee (Protocol No. 10‐12‐097). To generate tumour‐bearing mice, GK1 cells (0.5 × 10^6^) were subcutaneously inoculated into WT and *Pdia4^–/–^* mice on the B6 and *Rag1^–/‐^* backgrounds as published.^16^ Alternatively, GK1 cells (0.5 × 10^6^) were subcutaneously inoculated into *Rag1^–/‐^* mice. The mice were adoptively transferred with T (1.5 × 10^7^) and B (1.5 × 10^7^) cells, once every 3 days, for 28 days. Mouse tumour volume, tumour weight, survival and metastasis were regularly measured at the indicated time. Tumour volume (cm^3^) was measured with a caliper and estimated with the formula V = ½ × (*a* × *b* ^2^), where *a* and *b* corresponded to the longest and shortest diameters of the tumour, respectively. For lung metastasis, GK1 tumour‐bearing mice were sacrificed 40 days post‐cell injection. Their lungs were removed, examined for metastasis and analysed using Maestro EX Imaging System based on the manufacturer's protocol (Caliper, MA, USA). The number of lung nodules was counted.

### 2.4 Isolation and flow cytometric analysis of stromal cells

To isolate and analyse stromal cells, tumour tissues from WT and *Pdia4^–/–^* mice bearing GK1 tumours were removed and digested with digestion buffer (4 ml of 2.5% Collagenase l, 2 ml of 0.05% trypsin and 0.1 ml of DNase in 16 ml serum‐free RPMI medium) in an incubator at 37°C for 15 min. After washing, the cells were sorted using FACSAria cell sorter based on the presence of GFP. The purity of GFP‐positive GK1 cells (94%) and GFP‐negative stromal cells (99%) was used for further analysis. The GFP‐negative stromal cells were stained with antibodies against CD45, CD140a, CD31, B220, CD3, NKG2D, CD11b and Gr1 and analysed using an LSRII analyser. Alternatively, stromal cells were stained with isotope‐labeled antibodies against GFP, B220, CD3, CD140a, CD31, CD11b and Gr1 (Table S1), followed by CyTOF analyses (Fluidigm, CA, USA). All the flow cytometric data were processed using the FlowJo software with the SPADE plug‐in.

### 2.5 Functional assays of immune cells

To analyse lymphocyte proliferation, splenocytes and tumour‐infiltrating leukocytes of WT and *Pdia4^–/–^* mice bearing GK1 tumours were positively selected with microbeads coated with αCD19 or αCD4 antibody. B cells (10 000 cells/well) were stimulated with bacterial LPS and IL‐4 while T cells (30,000 cells/well) were stimulated with αCD3 and αCD28 antibody. After 3 days, the lymphocytes were pulsed with [^3^H]‐thymindine for 8 h. After extensive washing, the cells were harvested and counted using a β‐counter. For functional analysis of myeloid‐derived suppressor cells (MDSCs) for T_reg_ induction, CD11b^+^ Gr1^+^ MDSC (1 × 10^6^) isolated from the mice were co‐cultured with T cells (4 × 10^6^) from naïve mouse splenocytes. Four days later, the cells were stained with the antibodies against CD25 and Foxp 3 and analysed using an LSRII analyser.

### 2.6 Immunoblotting analysis

Total lysates of mouse skin stromal cells and cancer stromal cells (GFP^–^) and GFP^+^ GK1 cells from tumour tissues, and recombinant proteins underwent immunoblotting analysis as described.^16^ Jurkat cells were transfected with the indicated expression plasmids for domain mapping. After cell lysis, total lysates and their precipitates underwent immunoprecipitation and immunoblotting analysis. For protein stability test, Gst‐tagged Pdia4 (130 ng), Gst‐tagged Pdia4^280‐646^ (100 ng) and an irrelevant protein, bovine serum albumin (BSA), 90 ng, were incubated with an equimolar amount of His‐tagged Stat3 (120 ng) and phospho‐Stat3 (120 ng) in the presence of trypsin/Ethylenediaminetetraacetic acid (EDTA) (0.05%) for 10 min. The mixture underwent immunoblotting analysis using αHis and αGst antibodies.

### 2.7 Dual luciferase reporter assay

Jurkat and/or Raji cells were transfected with pStat3‐3×SBS‐TA‐FLuc or p3×SBS‐TA‐FLuc together with pβ‐Actin‐RLuc, Flag‐tagged Pdia4 and Flag‐tagged Pdia4^280‐646^ at different dosages. Ten micrograms of total lysates was subjected to the dual luciferase reporter assays (Promega, WI, USA). The efficiency of transfection, as determined by *Renilla* luciferase activity in the lysate, was used to normalize the activity of firefly luciferase. The normalized firefly luciferase activities are presented in arbitrary units as described previously.^20^

### 2.8 Cytokine antibody array

Mouse cytokine antibody arrays (C3 and C4, Raybiotech, GA, USA) were blocked and incubated with tumour conditioned medium, prepared from culture medium of GK1 cells. After extensive washing, the arrays were incubated with a biotinylated antibody cocktail and then streptavidin‐conjugated peroxidase. The arrays were developed with Chemiluminescent kits, photographed and quantified using ImageJ software (NIH, MD, USA).

### 2.9 Data processing and statistics

**FIGURE 1:**
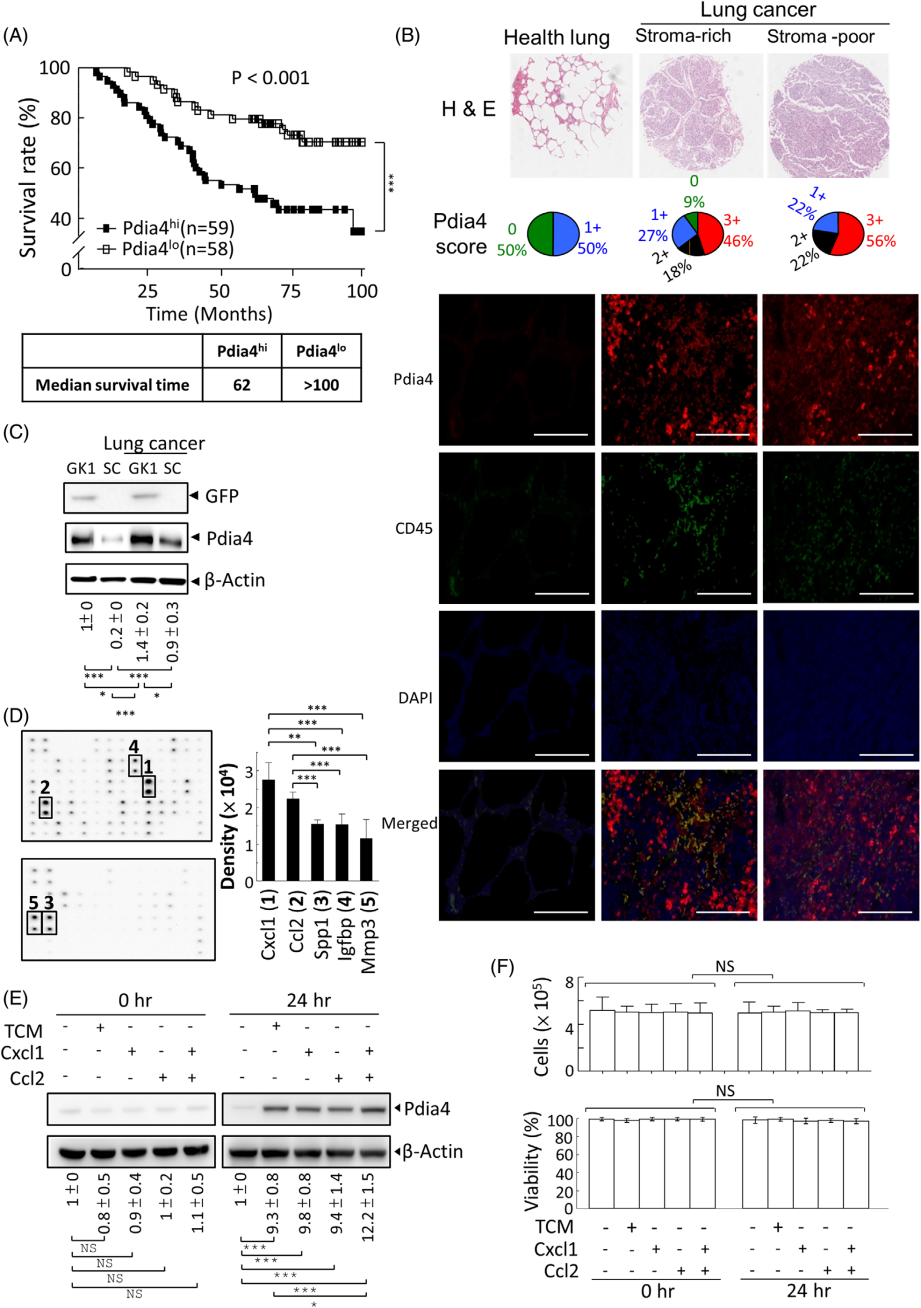
Pdia4 overexpression in lung cancer stroma of human and mouse origin and its correlation with patient survival. (A) Survival rate of the patients with lung cancer whose Pdia4 expression was high (Pdia4^hi^) and low (Pdia4^lo^) was obtained from the PrognoScan RNA database (GSE179339). Pdia4^hi^ and Pdia4^lo^ represented the signal of Pdia4 RNA in cancer tissues over and under 0.5 arbitrary units. (B) A human tumour tissue microarray (Biomax T045e) containing four normal lung tissues and 20 lung carcinomas (11 stroma‐rich and 9 stroma‐poor) was stained with Hematoxylin and eosin stain (HE) staining. Representative images (200×) from a tissue array are shown (1st row). The expression level of Pdia4 was scored, and the pie chart indicates the percentage of tissues on the array with scores of 0, 1, 2 and 3 (2nd row). Alternatively, the same tumour microarray was stained with DAPI and antibody against Pdia4 and CD45. Fluorescent images of the tissues are shown (3rd row) Scale bar: 100 μm. (C) The Pdia4 levels in GK1 cells, subcutaneous stromal cells (SC) of tumour‐free mice and GK1 cells of GK1 tumour‐bearing mice and cancer stroma (SC) of GK1 tumour‐bearing mice (lung cancer) were quantified using immunoblotting analysis with the indicated antibodies. The ratio of the Pdia4 signal to the β‐actin signal in each lane was calculated, and the ratio of the first lane was set as 1. Data from three immunoblots are presented as the mean ± SD. (D) A set of antibody arrays was used to quantify the level of 96 cytokines and soluble proteins in the tumour‐conditioned medium of GK1 cells. Representative blots are shown (left). Signal of top five proteins was measured and expressed as histograms (right). (E and F) Splenocytes of tumour‐free mice were treated with RPMI medium (CTR), tumour conditioned medium (TCM) or RPMI medium containing Cxcl1 (1 ng/ml), Ccl2 (0.5 ng/ml) and all for 0 h (left) and 24 h (right). One aliquot of the cells was lysed and analysed by immunoblotting analysis with the indicated antibodies (E). The ratio of the Pdia4 signal to the β‐actin signal in each lane was calculated using the same procedure (C). The other aliquot of the cells was counted for live and dead cells at 0 h and 24 h using trypan blue staining (F). The number of live cells (top) and cell viability (%, bottom) are shown. Data from three immunoblots are presented as the mean ± SD. GK1 cells were GFP‐expressing mouse lung cancer cells

Survival rate and median survival time in lung cancer patients were analysed and plotted based on the ProgScan RNA database. One (GSE13213) of the representative datasets was selected for survival rate analysis as shown in Figure 1A. The age range (32–84 years) and cancer stages (I: 56%, II: 15% and III: 29%) of the cohort (117 patients) were included. Data from three or more independent experiments are presented as mean ± standard deviation (SD). Comparisons between multiple groups were made with ANOVA. *p* values of less than 0.05 (*), 0.01 (**) and 0.001 (***) were considered to be statistically significant.

## 3 RESULTS

### 3.1 Pdia4 overexpression in the cancer stroma is inversely associated with survival in patients with lung cancer

First, we investigated the link between Pdia4 expression level and lung cancer in human clinical samples. Based on the PrognoScan RNA database (GSE13213, http://dna00.bio.kyutech.ac.jp/PrognoScan/index.html), patients with a high level of Pdia4 RNA (Pdia4^hi^) had a much lower survival rate than those with a low level of Pdia4 RNA (Pdia4^lo^) (top, Figure 1A). Accordingly, the median survival times in Pdia4^hi^ and Pdia4^lo^ patients were 62 and >100 weeks, respectively, which was clinically significant for lung cancer therapy (bottom, Figure 1A). The overall data indicated that the Pdia4 RNA level in lung cancer tissues was negatively correlated with survival of patients as shown by survival rate and median survival time (Figure 1A). Next, we examined the level of Pdia4 in cancer cells and cancer stromal cells in human lung cancer (Figure 1B). Pdia4 scores indicated that Pdia4 had a low expression level in healthy lung, and this expression was up‐regulated in lung cancer tissues irrespective of their stroma content (Pdia4 score, Figure 1B). To differentiate the Pdia4 expression in cancer stroma and lung cancer, we used anti‐CD45 antibody to stain stromal leukocytes. Immunohistochemical (IHC) staining data showed a basal protein level of Pdia4 and CD45 in lung tissues of healthy volunteers (Healthy lung, Figure 1B). In sharp contrast, Pdia4 and CD45 were highly expressed in lung cancer tissues compared to healthy lung (Pdia4 and CD45, Figure 1B). Of note, Pdia4 was highly expressed in CD45^+^ stromal leukocytes of human lung cancer as evidenced by the co‐localization of both molecules (Merged, Lung cancer, Figure 1B). In parallel, we checked the Pdia4 expression level in cancer cells and stromal cells of WT mice. Immunoblotting data showed that subcutaneous stromal cells, isolated from non‐tumour bearing WT mice, had a lower level of Pdia4 than GK1 cancer cells grown in culture (SC versus GK1, Figure 1C). In contrast, this expression was further up‐regulated in the cancer stromal cells of WT mice bearing GK1 cells, a mouse lung cancer line (SC/Lung cancer, Figure 1C). Antibody arrays were used to assess the level of 96 cytokines and serum proteins in the tumour‐conditioned medium (TCM) of GK1 cells. The data showed that Cxcl1, Ccl2, Spp1, Igfbp6 and Mmp3 were more abundant than the other proteins (Figure 1D). To decode the regulation of Pdia4 expression, TCM, Cxcl1, Ccl2 and a combination of Cxcl1 and Ccl2 were incubated with splenocytes. Co‐culture assays indicated that after 24 h treatment, Cxcl1, Ccl2 and both could induce a comparable level of Pdia4 level in splenocytes as TCM (Figure 1E). However, this induction was not ascribed to the change in cell proliferation and viability (Figure 1F).

### 3.2 Host Pdia4 promotes tumour development in mice with lung cancer and other cancer types

**FIGURE 2:**
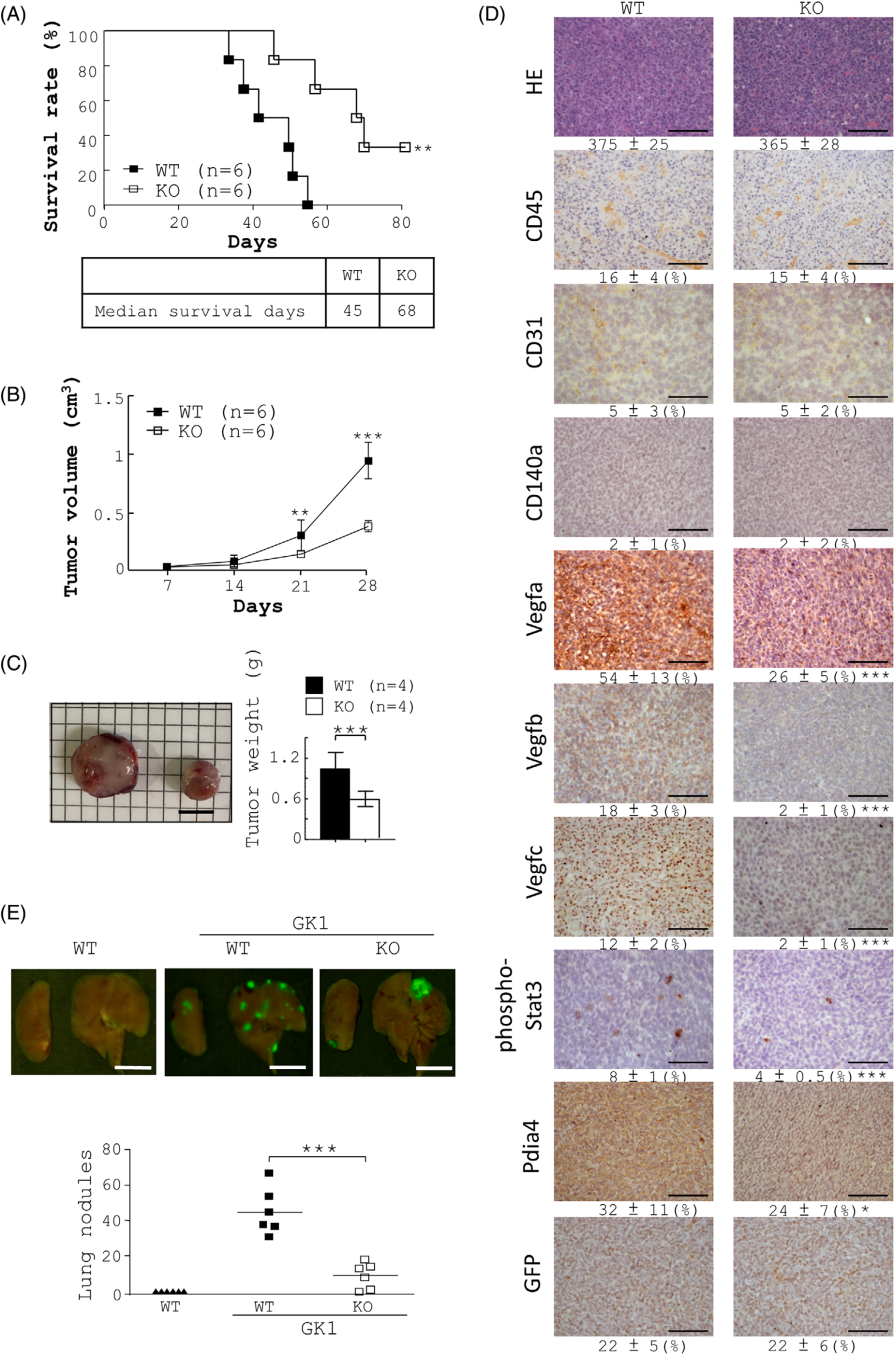
Effect of host Pdia4 on cancer development in GK1 tumour‐bearing mice. (A‐C) GK1 cells were subcutaneously injected into wild‐type (WT) and *Pdia4^–/–^* (KO) mice. Survival rate (A), tumour volume (B) and tumour weight (C) of the mice were measured at the indicated days post‐injection. The number (*n*) of mice is indicated. (D) Slides of the tumour tissues of the mice (A), which were sacrificed at 28 days post‐injection, were stained with HE and the indicated antibodies. Scale bar: 100 μm. (E) GK1 tumour‐bearing WT and *Pdia4^–/–^* (KO) mice were sacrificed 40 days after injection. Metastatic lungs were removed from the mice. GFP^+^ nodules in each mouse lung were detected using Maestro Imaging System as shown by green fluorescent signal. The number of green nodules was counted and re‐plotted into histograms. Scale bar: 1000 μm

Next, we studied the in vivo function of Pdia4 in tumour development using GK1 tumour‐bearing WT and *Pdia4^–/–^* mice. We found that *Pdia4^–/–^* mice bearing GK1 tumours had better survival than WT mice bearing GK1 tumours (Figure 2A). Median survival days in GK1 tumour‐bearing WT and *Pdia4^–/–^* mice were 45 days and 68 days, respectively. Consistently, GK1 tumours in *Pdia4^–/–^* mice had a smaller tumour volume (Figure 2B) and tumour weight (Figure 2C) than those in WT mice. Furthermore, IHC staining data showed that GK1 tumours in *Pdia4^–/–^* mice had a lower protein level of Vegfa, Vegfb, Vegfc and phosphao‐Stat3 than those in WT mice (Figure 2D). Consistently, WT mice bearing GK1 tumours had more lung metastasis than *Pdia4^–/–^* mice bearing GK1 tumours (Figure 2E). Similarly, *Pdia4^–/–^* mice bearing B16F10 and CT26 tumours had better survival than WT mice bearing B16F10 and CT26 tumours (Figure S1). Consistently, *Pdia4^–/–^* mice had a smaller tumour volume and a lower tumour weight than WT mice (Figure S1). Together, these data demonstrated that Pdia4 positively controlled tumour development in mice.

### 3.3 Host Pdia4 affects the number and function of stromal cells in mice bearing GK1 lung cancer

**FIGURE 3:**
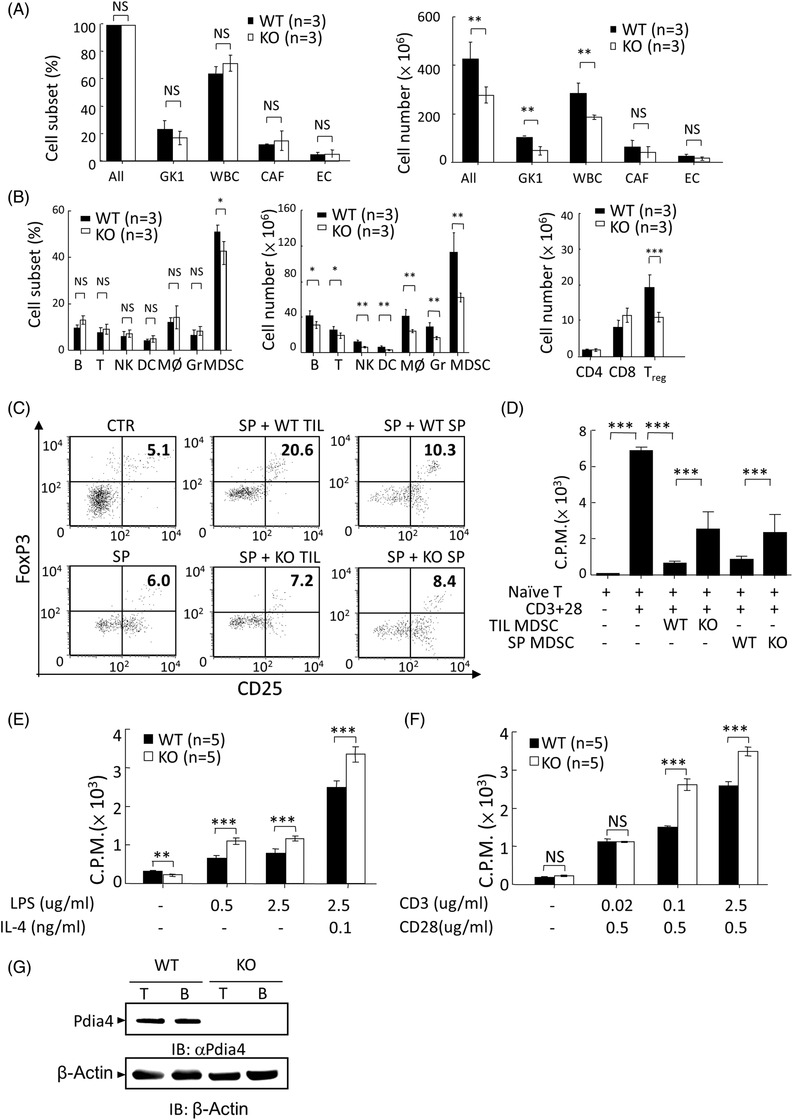
Effect of host Pdia4 on the composition and function of stromal cells in GK1 tumour‐bearing mice. **(A**) Wild‐type (WT) and *Pdia4^–/–^* (KO) mice bearing GK1 cells were sacrificed 28 days after injection. The tumours in both mouse groups were digested with proteases, sorted with GFP and counted. Total cells (ALL) denote the GK1 cells (GFP^+^ cells) and stromal cells (GFP^–^ cells) in mouse tumours. GFP^–^ cells were stained and analysed for CD45 (WBC), CD140a (CAF) and CD31 (EC) cells. The percentage (left) and number (right) of the above five subsets in the mouse tumours were plotted as histograms. (B) GFP^–^ cells were stained with αCD45 antibody plus αCD3 (T cells), αB220 (B cells), αGr1 (granulocytes, Gr), αNKG2D (NK cells), αCD11b (macrophages, MØ) and αCD11c (DC) antibodies, followed by flow cytometric analysis. CD45^+^CD11b^+^Gr1^+^ cells represent the myeloid‐derived suppressor cells (MDSCs) subset. The percentage (left) and number (middle) of the above seven subsets in the mouse tumours were plotted as histograms. Alternatively, GFP^–^ cells were stained with αCD3, αCD4, αCD8, αCD25, and αFoxP3 antibodies, followed by flow cytometry. The number of T‐cell subsets was analysed (right). (C) MDSC‐mediated T_reg_ induction was analysed. Naïve splenocytes were used as negative control (CTR). Naïve splenocytes were co‐cultured with Phosphate buffered saline (PBS) (SP), tumoural MDSC (SP + WT TIL) of WT mice bearing GK1 tumours, splenic MDSC (SP + WT SP) of WT mice bearing GK1 tumours, tumoural MDSC (SP + KO TIL) of Pdia4^–/–^ mice bearing GK1 tumours, and splenic MDSC (SP + KO SP) of Pdia4^–/–^ mice bearing GK1 tumours, in the presence of αCD3 plus αCD28 antibodies. After 4 days, the cells were stained with the indicated antibodies and analysed using a flow cytometer. (D) Naïve splenic T cells were incubated with MDSC of tumour sites and spleens from WT and *Pdia4^–/–^* (KO) mice, in the presence of αCD3 plus αCD28 antibodies, for 3 days. T cell proliferation was tested. (E‐F) Tumour infiltrating B cells (E) and T cells (F) of the mice (A) were activated with the indicated stimuli and tested for lymphocyte proliferation. (G) The level of Pdia4 in T and B cells purified from WT and *Pdia4^–/–^* (KO) mice bearing GK1 tumours was analysed with immunoblotting analysis. The number (*n*) of mice is indicated

To explore the type and function of stromal cells that host Pdia4 affects, we compared the cell composition and function of tumour stromal cells in WT and *Pdia4^–/‐^* mice bearing GK1 tumours. Flow cytometric data showed that GK1 tumour‐bearing WT mice had a similar composition of stromal cells and GK1 cells at the tumour site in both the groups of mice (left, Figure 3A). A closer analysis of stromal cells showed that GK1 tumour‐bearing WT mice had a slightly higher proportion of MDSC in tumour sites than GK1 tumour‐bearing *Pdia4^–/–^* mice (left, Figure 3B). However, GK1 tumour‐bearing WT mice had more GK1 cells and leukocytes in tumour sites than GK1 tumour‐bearing *Pdia4^–/–^* mice (right, Figure 3A). A closer analysis of stromal cells showed that GK1 tumour‐bearing WT mice had more MDSC, B cells, T cells and other types of leukocytes at tumour sites than GK1 tumour‐bearing *Pdia4^–/–^* mice (middle, Figure 3B). In addition, GK1 tumour‐bearing WT mice had more T_reg_ cells than GK1 tumour‐bearing *Pdia4^–/–^* mice (right, Figure 3B). However, CD4^+^T and CD8^+^T cells in both the groups of mice appeared to be similar (right, Figure 3B). To further understand the role of Pdia4 in cancer‐associated leukocytes, MDSC, T cells and B cells at tumour sites were isolated and tested for their activities. We found that the percentage of T_reg_ cells, CD25^+^FoxP3^+^ cells, in the spleen of naive mice and αCD3/αCD28‐activated splenocytes were 5.1% (Control (CTR), Figure 3C) and 6% (Spleen (SP), Figure 3C), respectively. This percentage was further elevated by MDSC. Tumoural MDSC, isolated from WT mice bearing GK1 cells, induced more T_reg_ cells than those, isolated from *Pdia4^–/–^* mice bearing GK1 cells (SP + WT TIL [20.6%] vs. SP + KO TIL [7.2%], Figure 3C). Similarly, splenic MDSC, isolated from WT mice bearing GK1 cells, induced more T_reg_ cells than those, isolated from *Pdia4^–/–^* mice bearing GK1 cells (SP + WT SP [10.3%] vs. SP + KO SP [8.4%], Figure 3C). We also examined the suppressive activity of MDSC using T cell proliferation as a readout. As a result, tumoural and splenic MDSC of WT mice suppressed T cell proliferation to a greater extent than tumoural and splenic MDSC of *Pdia4^–/–^* mice (WT TIL MDSC and WT SP MDSC vs. KO TIL MDSC and KO SP MDSC, Figure 3D). Furthermore, tumoural T (Figure 3E) and B cells (Figure 3F) of WT mice bearing GK1 cells had less proliferation than those of *Pdia4^–/–^* mice bearing GK1 cells. Accordingly, splenic B (Figure S2A) and T cells (Figure S2B) of WT mice bearing GK1 cells had less proliferation than those of *Pdia4^–/–^* mice bearing GK1 cells. The protein level of Pdia4 in purified T and B cells is shown (Figure 3G). Overall, the functional studies indicated that Pdia4 promoted MDSC‐mediated T_reg_ formation (Figure 3C), MDSC activity (Figure 3D) and inhibited activation of B and T cells (Figure 3E,F and Figure S2A,B). However, there was no difference in the composition of cell subsets in the bone marrow, thymus and spleen between WT and *Pdia4^–/–^* mice (Figure S3A). Moreover, there was no difference in the level of expression of Sdf‐1 and its chemokine receptor, Cxcr4, in bone marrow cells in WT and *Pdia4^–/–^* mice (Figure S3B,C). No difference in the proliferation of splenic B (Figure S3D) and T cells (Figure S3E) between WT and *Pdia4^–/–^* mice was observed. Overall, the data showed that Pdia4 increased the number of stromal leukocytes and their pro‐tumoural function.

### 3.4 T and B cells are required for host Pdia4‐mediated tumour development in mice bearing GK1 lung cancer

**FIGURE 4:**
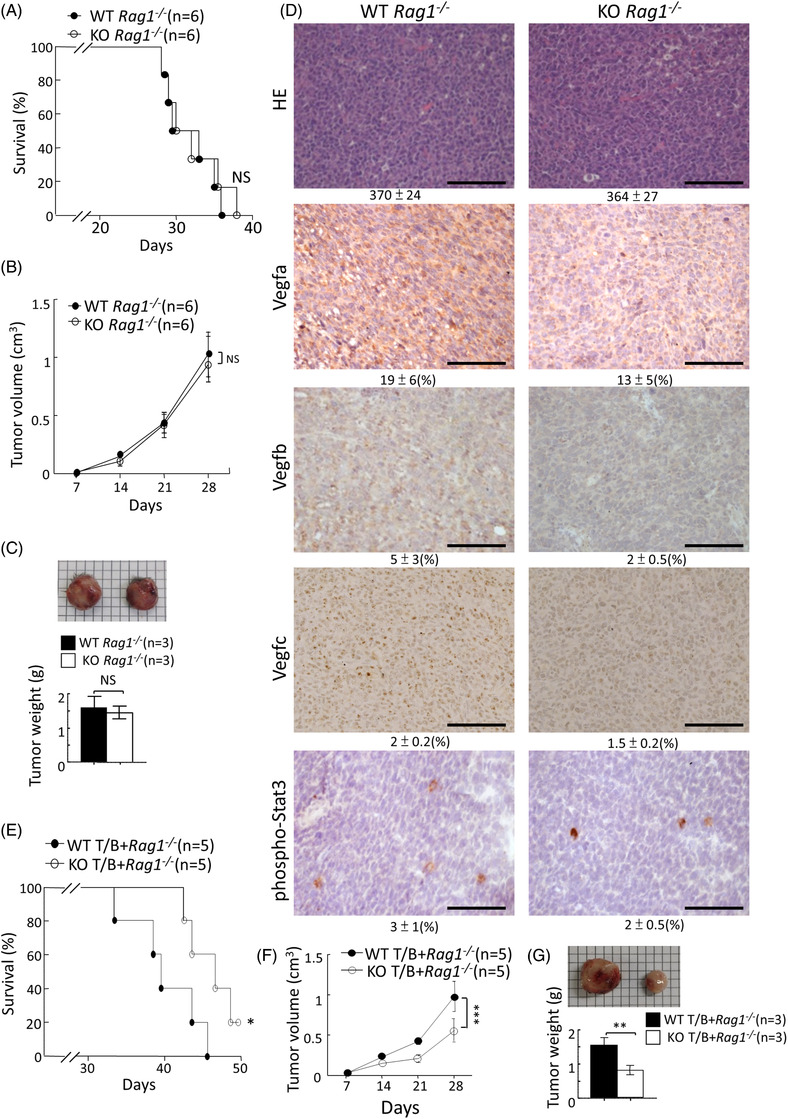
No difference in tumour development between wild‐type (WT) and *Pdia4^–/–^Rag1^–/–^* mice bearing GK1 cells. (A‐C) The same procedure as Figure 2A was conducted except that *Rag1^–/–^* (WT *Rag1^–/–^*) and *Pdia4^–/–^Rag1^–/–^* (KO *Rag1^–/–^*) mice were used. Survival rate (A), tumour volume (B) and tumour weight (C) of both groups were measured. (D) Slides of the tumour specimens of the mice 28 days after injection were stained with HE and antibodies against 3 Vegf proteins and phospho‐Stat3. Scale bar: 100 μm. (E‐G) The same procedure as Figure 2A was conducted except that *Rag1^–/–^* (WT *Rag1^–/–^*) mice were adoptively transferred with T and B cells of WT and *Pdia4^–/–^* mice. Their survival rate (E), tumour volume (F) and tumour weight (G) were monitored

Next, *Rag1^–/‐^* and *Rag1^–/‐^ Pdia4^–/‐^* mice bearing GK1 tumours were used to investigate the function of Pdia4 in T cell‐ and B cell‐implicated tumour development because T and B cells are deficient due to *Rag1* ablation. No difference in tumour development between GK1 tumour‐bearing *Rag1^–/–^* and *Rag1^–/–^Pdia4^–/–^* mice was noticed based on the survival rate (Figure 4A), tumour volume (Figure 4B) and tumour weight (Figure 4C). Accordingly, the activation of Stat3 and expression of Vegfa, Vegfb, and Vegfc in GK1 tumours between *Rag1^–/‐^* and *Rag1^–/‐^ Pdia4^–/–^* mice were not altered (Figure 4D). Moreover, no difference in the cell number and composition of GK1 cells and stromal cells was observed in *Rag1^–/–^* and *Rag1^–/–^Pdia4^–/–^* mice (Figure S4). To ascertain whether Pdia4 in T and B cells play a major role in GK1 tumour growth, WT and *Pdia4^–/–^* T and B cell were adoptively transferred into *Rag1^–/‐^* mice. As a result, GK1 tumour‐bearing *Rag1^–/‐^* mice that received *Pdia4^–/–^* T and B cells had better survival than those that received WT T and B cells (Figure 4E). Accordingly, GK1 tumour‐bearing *Rag1^–/‐^* mice that received *Pdia4^–/–^* T and B cells had smaller tumour volume (Figure 4F) and tumour weight (Figure 4G) than those that received WT T and B cells. The data were consistent with the discovery showing that WT T and B cells were more immunologically suppressive than *Pdia4^–/–^* T and B cells in GK1 tumour‐bearing mice. Collectively, the data showed the importance of B and T cells for host Pdia4‐mediated lung cancer development.

### 3.5 Establishment of the regulatory link among Pdia4, Stat3 and the Vegf family in the cancer stroma using a systems biology approach

To tease out the likely mechanism of host Pdia4 for tumour development, Affymetrix Genechips (Mouse Genome 430 2.0 array) were used to compare the gene expression profile of the cancer stroma in WT and *Pdia4^–/–^* mice on a genome‐wide scale. Among 34 000 genes, 14 151 genes whose signal intensity was over 200 were selected for further analysis (GSE179339 and Figure S5A). Among 14 151 genes, 1488 genes had an expression ratio of WT and *Pdia4^–/–^* stromata (WT/ *Pdia4^–/–^*) by 2.4‐fold or more and 66 genes had a ratio of 0.4 or less (GSE179339 and Figure S5A). IPA was employed to predict the signaling pathway of 1554 genes (Figure S5A), resulting in the discovery of 14 pathways based on and *z*‐score (>4) (Table S2). The ovarian cancer pathway, composed of 26 candidate genes, stood out of the 14 pathways according to −log *p* value (>2.6) (Figure S5A). Microarray data showed that WT stromata had a higher expression level of *Akt3*, *Ccnd1*, *Egfr*, *Fgfr1*, *Fzd6*, *Gja1*, *Irs1*, *Mras*, *Msh2*, *Msh6*, *Pa2g4*, *Pdgfc*, *Pgf*, *Pik3r4*, *Prkar2a*, *Prkar2b*, *Ptgs1*, *Rala*, *Rras2*, *Smo*, *Src*, *Tcf4*, *Tcf7l1*, *Vegfb*, *Vegfc* and *Wnt9a* than *Pdia4^–/–^* stromata (Figure S5B). However, the difference in the expression of *Egfr*, *Fgfr1*, *Fzd6*, *Mras*, *Pik3r4* and *Wnt9a* between WT and *Pdia4^–/–^* stromata was not statistically significant. Next, RT‐PCR data also confirmed that the difference in the expression level of the aforesaid 20 genes between WT and *Pdia4^–/–^* stromata was statistically significant (Figure S5C). In addition, WT stromata had a higher level of Vegfa than *Pdia4^–/–^* stromata (Figure S5C). However, the level of Stat3 in WT and *Pdia4^–/–^* stromata remained unaltered (Figure S5C). We also examined the protein level using immunoblotting analysis. The data showed that WT stroma had a higher level of Akt3, Ccnd1, Vegfa, Vegfb and Vegfc than *Pdia4^–/–^* stroma (Figure S5D,E). Moreover, WT stroma had a higher level of phosphorylated Stat3 (phospho‐Stat3) than *Pdia4^–/–^* stroma (Figure S5D,E). However, the level of Akt3 and Stat3 between WT and *Pdia4^–/–^* cancer stroma was slightly different (Figure S5D,E). In the interest of novelty and therapeutic potential in cancer stroma, we focused the remainder of this study on the link among Pdia4, Stat3 and the Vegf proteins in cancer stroma.

**FIGURE 5:**
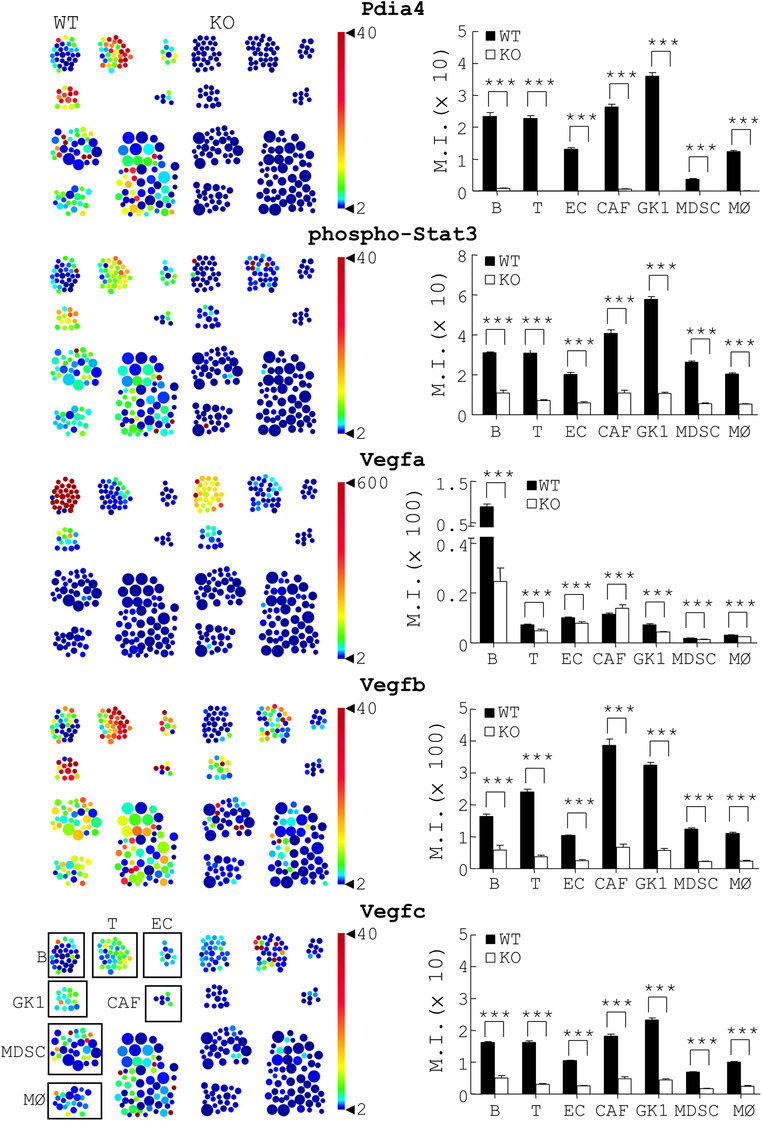
CyTOF analysis of Pdia4, phospho‐Stat3 and Vegf proteins in stromal subsets of GK1 tumour‐bearing wild‐type (WT) and *Pdia4^–/–^* mice. WT and *Pdia4^–/–^* (KO) mice bearing GK1 cells were sacrificed 28 days after injection. Tumour tissues were removed and digested with protease. The cells were stained with the indicated antibodies and analysed with a mass flow cytometer. The data were analysed with FlowJo software. SPADE analysis of GK1, B, T, EC, CAF, myeloid‐derived suppressor cell (MDSC), and MØ cells of WT and *Pdia4^–/–^* (KO) tumour sites was performed (right). The mean intensity (MI) of Pdia4, phospho‐Stat3 and 3 Vegf proteins in those cells was re‐plotted into histograms (right)

Next, mass flow cytometry (CyTOF) was used to confirm the protein level of Pdia4, Vegfa, Vegfb, Vegfc and phospho‐Stat3 in stromal subsets of GK1 tumour‐bearing WT and *Pdia4^–/–^* mice (Figure 5). As expected, CyTOF data showed that Pdia4 was only expressed in the stromal cells of GK1 tumour‐bearing WT but not *Pdia4^–/–^* mice (1st row, Figure 5). In contrast, phospho‐Stat3, Vegfa, Vegfb and Vegfc were differentially expressed in GK1 stromal cells as well as cancer cells (2nd to 5th rows, Figure 5). Of note, Vegfa was mainly expressed in B lymphocytes. Furthermore, WT cancer stroma had a higher level of phospho‐Stat3, Vegfa, Vegfb and Vegfc than those in *Pdia4^–/–^* stroma (2nd to 5th rows, Figure 5). Overall, the data suggest a regulatory link among Pdia4, Stat3 and the Vegf family in B cells, T cells and other stromal cells.

### 3.6 Molecular regulation of the Stat3‐mediated Vegf expression in stromal lymphocytes by Pdia4

**FIGURE 6:**
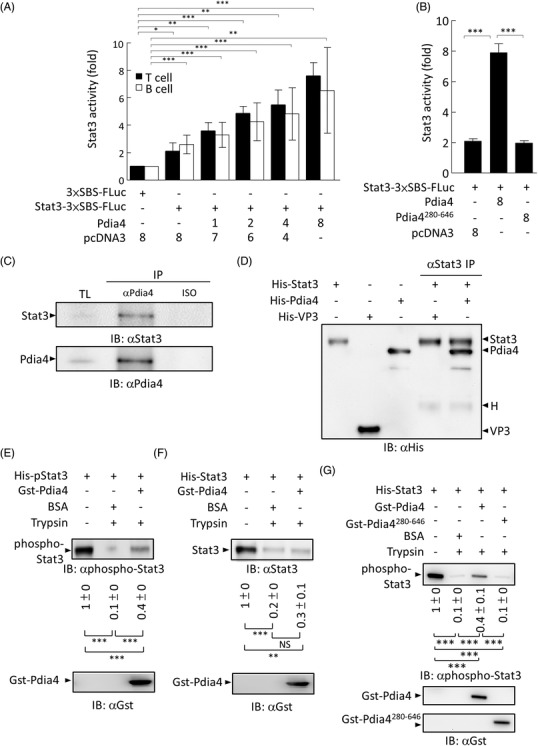
Pdia4 modulates Stat3 activation via the stabilization of Stat3 and its active form. (**A**) Jurkat cells and Raji cells were transfected with pβ‐Actin‐RLuc, an expression vector encoding Flag‐tagged Pdia4 at different amounts, p3×SBS‐FLuc and/or pStat3‐3×SBS‐FLuc. After 24 h, the cells underwent lysis and dual luciferase assays. pcDNA3 was used as a plasmid control to assure the use of an equal amount (8 μg) of plasmid DNA in each group. (B) The same procedure was performed in Jurkat cells except that a plasmid encoding Flag‐tagged Pdia4^280‐646^ was used. (C) After lysis, total lysates of Jurkat cells were incubated with αPdia4 or isotype (ISO) antibodies plus protein G beads. Their total lysates (TL) and immunoprecipitates (IP) underwent immunoblotting analysis using antibodies against Pdia4 (αPdia4) and Stat3 (αStat3). (D) His‐tagged Stat3 was in vitro incubated with His‐tagged Pdia4 or His‐tagged VP3, an irrelevant protein. After αStat3 precipitation, recombinant proteins and their immunoprecipitates (IP) underwent immunoblotting analysis using αHis antibody. H indicates a heavy chain of antibody. (E‐F) Gst‐tagged Pdia4 or BSA was incubated with an equimolar amount of His‐tagged phospho‐Stat3 (E) and Stat3 (F) in the presence of trypsin plus EDTA. The mixture underwent immunoblotting analysis using the indicated antibodies. (G) Gst‐tagged Pdia4, Gst‐tagged Pdia4^208‐646^ or BSA was incubated with an equimolar amount of His‐tagged phospho‐Stat3 in the presence of trypsin plus EDTA, followed by immunoblotting analysis

To study the molecular mechanism by which Pdia4 regulated Stat3 and the Vegf family in cancer stroma, we first examined the relationship between Stat3 and Pdia4. We found that Pdia4 enhanced Stat3‐mediated transcriptional activity in Jurkat cells and Raji cells using dual luciferase assays (Figure 6A). In marked contrast, a truncated mutant of Pdia4 that lost its ability to bind Stat3, Pdia4^280‐646^ failed to enhance this transcription in Jurkat cells (Figure 6B). Accordingly, co‐expression of Pdia4 and Stat3 in Jurkat cells indicated that Pdia4 interacted with Stat3 in vivo (Figure 6C). Further, co‐incubation of recombinant Pdia4 and Stat3 demonstrated a direct interaction between Pdia4 and Stat3 in vitro (Figure 6D). We also explored the action of Pdia4 on the stability of Stat3 and phospho‐Stat3 in the presence of protease. The co‐incubation assays showed that Pdia4 inhibited the trypsin‐mediated degradation of phospho‐Stat3 better than bovine serum albumin, an irrelevant protein, (Figure 6E). This inhibition of the trypsin‐mediated degradation of Stat3 was similar but not statistically significant (6F). However, Pdia4^280‐646^ lost this inhibition (Figure 6G). Apparently, the first two CGHC motifs (a.a. 1–279) of Pdia4 were important for Stat3 function.

**FIGURE 7:**
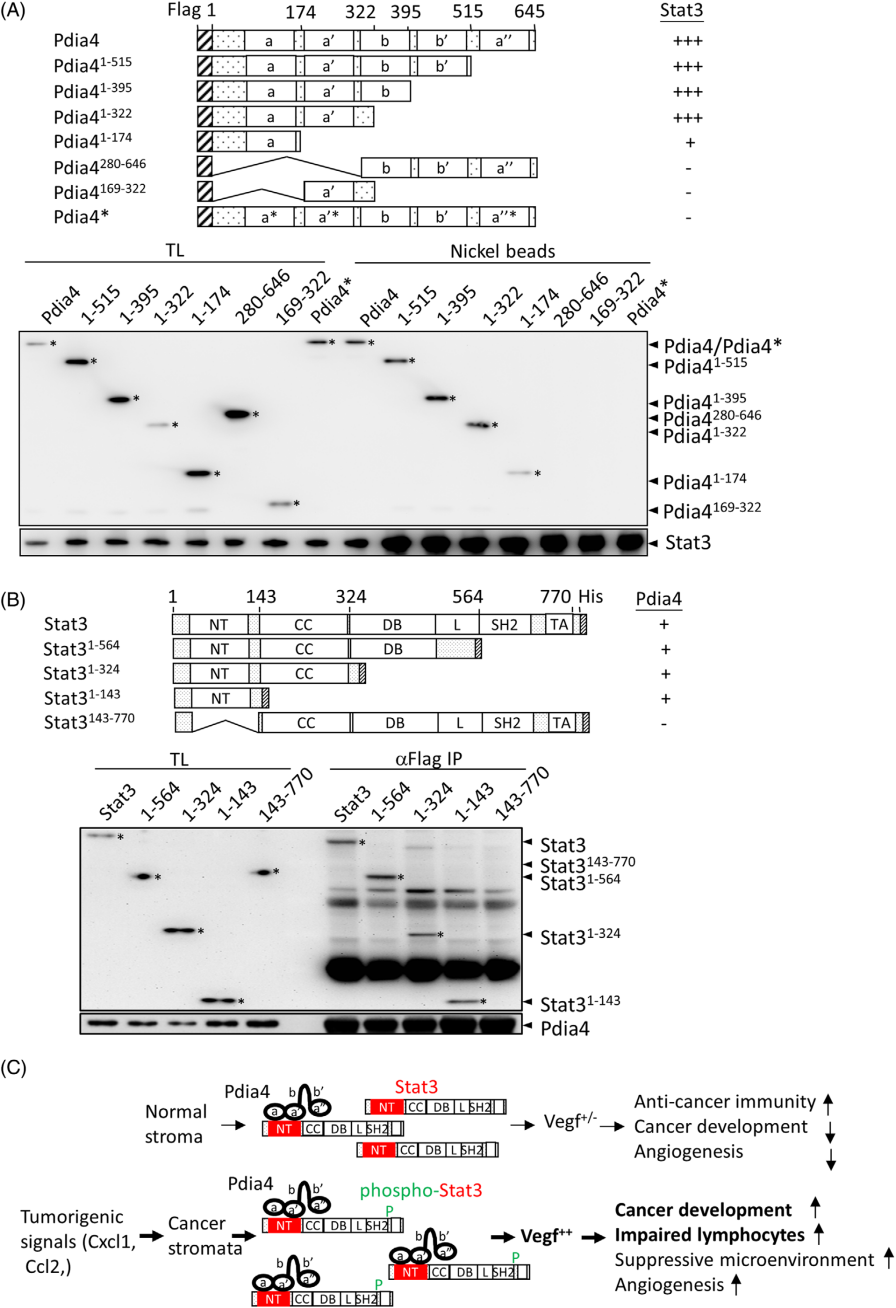
Mapping of the interaction domain between Pdia4 and Stat3. (A) The construct encoding Flag‐tagged Pdia4 or its mutants and that expressing His‐tagged Stat3 were co‐transfected into Jurkat cells. Their total lysates (TL) and precipitates of nickel beads underwent immunoblotting analysis. Pdia4 and Stat3 were visualized with Flag antibodies (αFlag, top) and His (αHis, bottom). (B) The construct encoding His‐tagged Stat3 or their mutants and that expressing Flag‐tagged Pdia4 were co‐transfected into Jurkat cells. Their total lysates (TL) and αFlag immunoprecipitates (IP) underwent immunoblotting analysis using αHis (top) or αFlag (bottom) antibodies. (C) A schema illustrating the regulation of Stat3 and its downstream molecules, Vegf proteins, by Pdia4 via a CGHC‐dependent intermolecular interaction in cancer stroma. In response to tumourigenic signals such as Cxcl1 and Ccl2, Pdia4 interacts with Stat3 and its active form, phospho‐Stat3. This interaction leads to phospho‐Stat3‐mediated Vegf expression. As a result, increased Vegf family proteins inactivate lymphocytes in cancer microenvironment and, in turn, promote cancer development

Next, to pinpoint the interaction domain in Pdia4 and Stat3, Jurkat cells co‐expressing full‐length Stat3 with His tag at its C‐terminus and Pdia4 or its deletion/point mutation mutants with Flag tag at their N‐terminus were used (Figure 7). Co‐immunoprecipitation with nickel beads demonstrated that the first two CGHC domains of Pdia4 were mainly responsible for its interaction with Stat3 (Figure 7A). Further, when CGHC was converted into cysteine‐to‐serine mutations in thioredoxin domains (SGHS), a tetrapetide of serine‐glycine‐histidine‐serine, in the three CGHC domains of Pdia4, it lost its ability to bind Stat3 (Pdia4*, Figure 7A). The same strategy was used to characterize the interaction domain of Stat3 for Pdia4. The N‐terminal domain corresponding to a. a. 1–143 of Stat3 (Stat3^1‐143^) was found to be the domain for Pdia4 association (Figure 7B). Together, these results suggested that Pdia4 used its first two CGHC domains to interact with the N‐terminal domain of Stat3.

Since host Pdia4 in T and B lymphocytes was pivotal for lung cancer development (Figures 2 and 4), we then addressed how Pdia4 controlled Stat3‐mediated expression of the Vegf family in both lymphocytes (Figure S6). Co‐transfection of the expression plasmid of Pdia4 and Stat3 with the reporter construct composed of the *Vegf* promoters indicated that Pdia4 increased the Stat3‐mediated transcription of *Vegfa, Vegfb and Vegfc* gene in Raji cells (Figure S6A) and Jurkat cells (S6B). Interestingly, Stat3 could dose‐dependently augment the expression of Pdia4 (left, Figure S6C). This expression was further increased by Cxcl1 and Ccl2 (right, Figure S6C). However, a dominant‐negative mutant of Stat3, Stat3Y750F, reduced Pdia4 transcription mediated by Cxcl1 and Ccl2 (right, Figure S6C). The data implied a feedforward relationship between Pdia4 and Stat3. On the other hand, functional studies indicated that Vegfa, Vegfb and Vegfc compromised the activation of B and T cells (Figure S6D). In parallel, Matrigel plug assays showed that WT mice had more angiogenesis than *Pdia4^–/‐^* mice (CTR, Figure S7). This difference in the angiogenesis between WT and *Pdia4^–/‐^* mice was further increased by Vegfa (Vegfa, Figure S7). Overall, this study supported the notion that Pdia4 regulated Sta3‐mediated pathways involving the Vegf family in lymphocytes. The data also demonstrated the mechanism of Pdia4 in the cancer stroma and its potential for use as a molecular target for cancer therapy.

Under physiological conditions, normal lung stromata express an inactive form of Stat3 and a basal level of Pdia4 and, in turn, a basal level of Vegf proteins (Figure 7C). During tumourigenesis, tumourigenic signals such as Cxcl1 and Ccl2 induce Pdia4 overexpression and Stat3 phosphorylation and, subsequently, lead to an up‐modulation of Vegf proteins in lung cancer stromata. As a result, Vegf promotes cancer development via induction of suppressive cancer microenvironment (impaired lymphocytes and suppressive immune cells) as well as angiogenesis (Figure 7C).

## 4 DISCUSSION

Pdia4 was initially identified as a key regulator of cancer to increase cell growth through inhibition of the procaspase pathway in cancer cells.^16^ In this study, we extended our research to investigate its significance in cancer stroma. We first confirmed that the Pdia4 level in lung cancer and other cancer types (brain cancer, renal carcinoma, melanoma and colorectal cancer) was inversely correlated to patient survival (Figure 1 and Figure S8). Strikingly, Pdia4 reduction in lung cancer prolonged the median survival time by 38 months, which was very significant for lung cancer patients (Figure 1A). For the first time, we showed that Pdia4 expression was induced in lung cancer stroma of human and mouse origin (Figure 1). We also found that Pdia4 activated Stat3 and its downstream pathways (e.g., increased expression of Vegf family), leading to a promotion of lung cancer development in mice. Apparently, the Pdia4/Stat3/Vegf axis in T and B lymphocytes seemed to be important for lung cancer development. The novel cascade in stromal cells facilitated the formation of immunosuppressive cancer microenvironment that favored cancer development. In sharp contrast, the Pdia4/procaspases 3/7 pathway in cancer cells also favoured cancer development.^16^ Although the possibility that the Pdia4/Stat3/Vegf axis also functions in cancer cells could not be excluded, both lines of evidence suggest that targeting Pdia4 in the cancer stroma and cancer cells is a promising two‐pronged approach to treating lung cancer.

Genetics studies in WT and *Pdia4^–/–^* mice with lung cancer and other cancers (melanoma and colorectal cancer) showed that host Pdia4 in the cancer stroma regulated cancer development (Figure 2 and Figure S1). This regulation was through stromal subsets, particularly T and B lymphocytes (Figure 4 and Figure S6). Cytokines in tumour stroma are thought to be key factors for the change in the composition and function of stromal cells. We confirmed that Cxcl1 and Ccl2 were the two mediators present in tumour conditioned medium of GK1 cells (Figure 1D). Further, both the mediators per se and in combination enhanced the expression of Pdia4 in stromal cells (Figure 1D). Furthermore, we also elucidated the mechanism by which Pdia4 exerted its action to facilitate lung cancer development. To date, the contribution of tumour Pdia4 versus host Pdia4 in tumour development has been poorly studied. A systems biology approach suggested that Pdia4 activates Stat3 and its downstream pathway in cancer stroma (Figure 5 and Figure S5). Since Pdia4 and Stat3 were present in stromal cells and tumour cells, CyTOF data showed that WT stroma had a higher level of phospho‐Stat3, Vegfa, Vegfb and Vegfc than *Pdia4^–/–^* stroma (Figure 5). Furthermore, we established a direct link between Pdia4, Stat3 and Vegf family in cancer stroma. This work, therefore, identified Pdia4 as an important player in cancer stroma, thus rendering Pdia4 a promising therapeutic target for cancer stroma.

Pdia4 is known to exert different functions by interaction with its specific partners/substrates. However, the partners and/or substrates of Pdia4 are largely unknown, and even less is known about its tumour promotion mechanism. Our previous publication reported that Pdia4 acted as a chaperone to control the activation/degradation of procaspases 3 and 7 and, in turn, cancer cell growth.^16^ Here, we addressed the intermolecular interaction between Pdia4 and Stat3 in cancer stroma. Our data showed that Pdia4 could interact with Stat3 (Figure 7). This interaction involved the stabilization of phospho‐Stat3 in the presence of protease (Figure 6). Moreover, domain mapping analysis of Pdia4 and Stat3 suggested Pdia4 used its first 2 CGHC domain to interact with the N‐terminal domain of Stat3. Overall, the data added Stat3 to a list of partners and substrates of Pdia4. Although Pdia4 has an ER retention signal, ER retention motif (KEEL) at its C‐terminal, our and other groups have reported that Pdia4 was situated in the cytosol, nucleus and membrane of cells as well as blood plasma.^16^, ^20^ Therefore, it is not hard to imagine that Pdia4 can directly interact with Stat3 in the cytosol and nucleus of cancer stromal cells, leading to the up‐regulation of *Vegf* gene expression (Figure S6). In the BioGPS database, it is reported that Vegfa (http://biogps.org/#goto = genereport&id = 7422), Vegfb (http://biogps.org/#goto = genereport&id = 7423) and Vegfc (http://biogps.org/#goto = genereport&id = 7424) are expressed in some stromal cells. The Vegf family has been reported to exert its action to suppress the function of immune cells.^12^, ^13^ Consistently, we discovered that Vegfa, Vegfb and Vegfc inhibited the activation of T and B cells (Figure S6D). Consequently, the Pdia4/Stat3 axis increased the Vegf family in stromal cells and, in turn, assisted in creating an immunosuppressive cancer microenvironment as well as angiogenesis, leading to cancer development (Figure 7C and Figure S7). CD31 in WT and Pdia4^–/‐^ mice bearing GK1 tumours was low (5%–10%) in the IHC (Figure 2D) and flow cytometry data (Figure 3A). Moreover, no difference in the expression level of CD31 between WT mice bearing GK1 tumours and Pdia4^–/‐^ mice bearing GK1 tumours was seen. However, the Matrigel plug assays clearly demonstrated that Pdia4 positively regulated angiogenesis in mice (Figure S7). Our data therefore argued that CD31 was a reliable marker of angiogenesis.

## CONFLICT OF INTEREST

The authors declare no conflict of interest.

## Supporting information

Supporting Information

## Data Availability

The data that support the findings of this study are available from the corresponding author upon reasonable request.
