# Supplementary material for: Protein disulfide isomerase a4 promotes lung cancer development via the Stat3 pathway in stromal cells
Source: Clin Transl Med. 2022 Feb 15;12(2):e606. doi: 10.1002/ctm2.606 (PMC8847735; doi:10.1002/ctm2.606)
Supplement: Supplementary file 1 — Supporting Information [file CTM2-12-e606-s001.docx]

**Supporting Information**

**Protein disulfide isomerase a4 promotes lung cancer development via the Stat3 pathway in stromal cells**

Tzung-Yan Chen^1,2^, Chun-Yen Yang^1,3^, Meng-Ting Yang^1^, Tien-Fen Kuo^1^, Cicero Lee-Tian Chang^4^, Chih-Li Chen^1^, Ching-Feng Chiu^7^, Yang-Hao Yu^,8^, Tsung-Han Lee^3^, Greta Yang^1^, and Wen-Chin Yang^1,2,3,5,6*^

^1^Agricultural Biotechnology Research Center, Academia Sinica, Taipei, Taiwan

^2^Institute of Biotechnology, National Taiwan University, Taipei, Taiwan

^3^Life Sciences, National Chung Hsing University, Taichung, Taiwan

^4^Department of Veterinary Medicine, National Chung-Hsing University, Taichung, Taiwan

^5^Institute of Pharmacology, National Yang-Ming University, Taipei, Taiwan

^6^Department of Aquaculture, National Taiwan Ocean University, Taipei, Taiwan

^7^Graduate Institute of Metabolism and Obesity Sciences, College of Nutrition, Taipei Medical University, Taipei, Taiwan

^8^Divisions of Pulmonary and Critical Care Medicine, Department of Internal Medicine, China Medical University Hospital, Taichung, Taiwan

**Corresponding author:**

Wen-Chin Yang, Agricultural Biotechnology Research Center, 128, Sec. 2, Academia Road, Nankang, Taipei 115, Taiwan. Tel: 886-2-27872076; Fax: 886-2-27822245; E-mail: wcyang@gate.sinica.edu.tw

**Running title:** Pdia4/Stat3 axis and lung cancer stroma

**Keywords:** Pdia4, lung cancer, Stat3, cancer stroma

**Table S1. A list of antibodies used in the study.**

| SN | Antibody | Vendor | Catalogue No. | Conjugate |
| --- | --- | --- | --- | --- |
| 1 | Anti-Pdia4 | Enzo | ADI-SPS-720-F | - or ^159^Tb |
|  |  | BD Biosciences | 610970 | - |
| 2 | Anti-Actin | Sigma-Aldrich | MAB1501 | - |
| 3 | Anti-Mouse Ig | Sigma-Aldrich | SAB3701095 | Peroxidase |
|  |  | Thermo Fisher | A10521 | Cy3 |
| 4 | Anti-Rabbit Ig | Sigma-Aldrich | A4914 | Peroxidase |
|  |  | Thermo Fisher | F-2765 | FITC |
|  |  | Fluidigm | 3175002G | ^175^Lu |
| 5 | Anti-Rat Ig | Sigma-Aldrich | A5795 | Peroxidase |
|  | Anti-Goat IgG | Sigma-Aldrich | A4174 | Peroxidase |
| 6 | Anti-CD45 | BD Biosciences | 559864 | APC |
|  |  | Fluidigm | 3147003C | ^147^Sm |
|  |  | Bioss Inc | bs-0522R | - |
| 7 | Anti-CD31 | BD Biosciences | 562861 | PerCP-Cy5.5 |
|  |  | Fluidigm | 3165013B | ^165^Ho |
| 8 | Anti-VEGFa | Santa Cruz | sc-152 | - |
| 9 | Anti-VEGFb | R&D Systems | MAB751 | - or ^155^Gd |
| 10 | Anti-VEGFc | Thermo Fisher | 34-5500 | - or ^161^Dy |
| 11 | Anti-Stat3 | Cell Signaling | 9139 | - |
| 12 | Anti-phospho-Stat3 (Tyr705) | Cell Signaling | 9145 | - |
|  |  | Fluidigm | 3158005A | ^158^Gd |
| 13 | Anti-GFP | Abcam | ab6662 | FITC |
| 14 | Anti-FITC | Fluidigm | 3174006B | ^174^Yb |
| 15 | Anti-CD140a | BD Biosciences | 562776 | PE |
| 16 | Anti-PE | Fluidigm | 3156005B | ^156^Gd |
| 17 | B220 antibody | BioLegend | 103208 | PE |
|  |  | Fluidigm | 3176002C | ^176^Yb |
| 18 | Anti-CD3 | BD Biosciences | 555276 | PE-Cy5 |
|  |  | BD Biosciences | 553057 | - |
|  |  | Fluidigm | 3152004C | ^152^Sm |
| 19 | Anti-CD4 | BioLegend | 100412 | APC |
| 20 | Anti-CD8 | BD Biosciences | 553031 | FITC |
| 21 | Anti-NKG2D | BD Biosciences | 558403 | PE |
| 22 | Anti-CD11b | BioLegend | 101208 | PE |
|  |  | Fluidigm | 3148003C | ^148^Nd |
| 23 | Anti-CD11c | BD Biosciences | 550261 | APC |
|  |  | Fluidigm | 31420003C | ^142^Nd |
| 24 | Anti-Gr1 | BD Biosciences | 553126 | FITC |
|  |  | Fluidigm | 3141005C | ^141^Pr |
| 25 | Anti-CD28 | BD Biosciences | 553295 | - |
| 26 | Anti-His Tag | Applied Biological Materials | G020 | - |
| 27 | Anti-Flag Tag | BioLegend | 637302 | - |
| 28 | Anti-Gst Tag | BioLegend | 640801 | - |
| 29 | Anti-Akt3 | Thermo Fisher | PA1-41700 | - |
| 30 | Anti-Ccnd1 | Thermo Fisher | 70421 | - |
| 31 | Anti-FoxP3 | eBioscience | 12-5773 | PE |
| 32 | Anti-CD25 | BD Biosciences | 552880 | PE-Cy7 |

**Table S2. IPA analysis of signaling pathways and their genes.**

| Ingenuity Canonical Pathways | -log(P-value) | z-score | Molecules |
| --- | --- | --- | --- |
| Ovarian Cancer Signaling | 2.96 | 4 | TCF4,RALA,PA2G4,VEGFB,PIK3R4,CCND1,PDGFC,PGF,SMO,MRAS,AKT3,EGFR,SRC,GJA1,WNT9A,FGFR1,PTGS1,PRKAR2A,VEGFC,TCF7L1,PRKAR2B,RRAS2,MSH2,IRS1,FZD6,MSH6 |
| Glioblastoma Multiforme Signaling | 2.58 | 4.796 | RALA,PDIA3,PDGFA,PIK3R4,CCND1,PDGFC,PLCD3,E2F6,PLCE1,RHOD,SOS1,MRAS,SMO,E2F5,PDGFRA,AKT3,E2F8,EGFR,SRC,RHOC,WNT9A,FGFR1,RRAS2,IRS1,ITPR3,E2F7,FZD6 |
| Thrombin Signaling | 2.1 | 4.2 | MPRIP,RALA,F2R,PDIA3,GNA11,PIK3R4,MAPK11,PTK2,ROCK2,GNB4,PLCD3,PLCE1,RHOD,SOS1,MRAS,AKT3,GNA13,GNB1L,PRKCA,EGFR,SRC,ARHGEF12,RHOC,FGFR1,ADCY6,MAPK12,RRAS2,PRKCI,IRS1,ITPR3 |
| Colorectal Cancer Metastasis Signaling | 1.95 | 4.382 | TCF4,RALA,VEGFB,MMP13,PIK3R4,PDGFC,CCND1,PGF,GNB4,RHOD,SOS1,SMO,MRAS,AKT3,MMP11,GNB1L,EGFR,SRC,RHOC,WNT9A,FGFR1,PRKAR2A,ADCY6,VEGFC,TCF7L1,MAPK12,PRKAR2B,RRAS2,MSH2,IRS1,MSH6,FZD6,TGFB3,TNF |
| Paxillin Signaling | 1.8 | 4.123 | SRC,PARVA,RALA,FGFR1,ITGA6,MAPK12,PIK3R4,MAPK11,BCAR1,PTK2,ITGA3,RRAS2,TLN2,IRS1,SOS1,MRAS,VCL,ACTN1 |
| GP6 Signaling Pathway | 1.31 | 4.243 | LAMA5,COL4A1,COL6A2,FGFR1,COL8A1,PIK3R4,COL16A1,COL5A1,PTK2,LAMC1,PRKCI,COL6A1,IRS1,LAMB1,AKT3,COL18A1,COL7A1,PRKCA |
| Role of NFAT in Cardiac Hypertrophy | 1.3 | 4.899 | RALA,PDIA3,CACNB3,PIK3R4,MAPK11,PLCD3,GNB4,PLCE1,SOS1,MRAS,AKT3,GNB1L,PRKCA,SRC,HDAC2,FGFR1,ADCY6,PRKAR2A,CACNA1C,MAPK12,PRKAR2B,PRKCI,RRAS2,IRS1,ITPR3,CACNA2D1,TGFB3,IL11 |
| IL-8 Signaling | 1.06 | 4.264 | SRC,RALA,RHOC,FGFR1,VEGFC,VEGFB,PIK3R4,MAPK12,CCND1,PDGFC,PGF,PTK2,ROCK2,GNB4,RRAS2,PRKCI,RHOD,IRS1,MRAS,AKT3,GNA13,GNB1L,EGFR,PRKCA |
| Integrin Signaling | 0.955 | 4.796 | SRC,PARVA,MPRIP,RALA,RHOC,FGFR1,ITGA6,PIK3R4,GIT1,BCAR1,ARHGAP5,PTK2,ITGA3,RRAS2,TLN2,RHOD,IRS1,SOS1,MRAS,CAV1,PFN2,AKT3,VCL,CTTN,ACTN1 |
| GNRH Signaling | 0.936 | 4.123 | SRC,RALA,GNA11,PRKAR2A,ADCY6,CACNA1C,CACNB3,MAPK12,MAPK11,PTK2,RRAS2,PRKCI,PRKAR2B,SOS1,ITPR3,CACNA2D1,MRAS,EGFR,PRKCA,MAP3K2 |
| Cardiac Hypertrophy Signaling | 0.775 | 4.796 | RALA,PDIA3,GNA11,PIK3R4,MAPK11,ROCK2,PLCD3,GNB4,PLCE1,RHOD,SOS1,MRAS,GNA13,GNB1L,MAP3K2,RHOC,FGFR1,ADCY6,PRKAR2A,CACNA1C,MAPK12,PRKAR2B,RRAS2,IRS1,TGFB3,HSPB1 |
| CREB Signaling in Neurons | 0.738 | 4 | RALA,PDIA3,FGFR1,GNA11,PRKAR2A,ADCY6,CACNA1C,CACNB3,PIK3R4,PLCD3,GNB4,RRAS2,PRKAR2B,PLCE1,PRKCI,IRS1,SOS1,ITPR3,CACNA2D1,MRAS,AKT3,GNA13,GNB1L,PRKCA |
| Signaling by Rho Family GTPases | 0.627 | 4.359 | CDC42EP5,GNA11,PIK3R4,PTK2,ROCK2,GNB4,ITGA3,CFL2,RHOD,EZR,MRAS,GNA13,GNB1L,CDH13,ARHGEF12,RHOC,FGFR1,RDX,WASF1,ARHGEF17,MAPK12,PRKCI,IRS1,MAP3K20,ARHGEF18,PARD3 |
| Dopamine-DARPP32 Feedback in cAMP Signaling | 0.538 | 4 | PDIA3,PPP2R2A,PRKAR2A,ADCY6,CACNA1C,PAWR,PRKG2,PLCD3,PRKCI,PLCE1,PRKAR2B,PPP2R3A,PPM1L,ITPR3,PPP2R5E,CAMKK2,PRKCA |

**
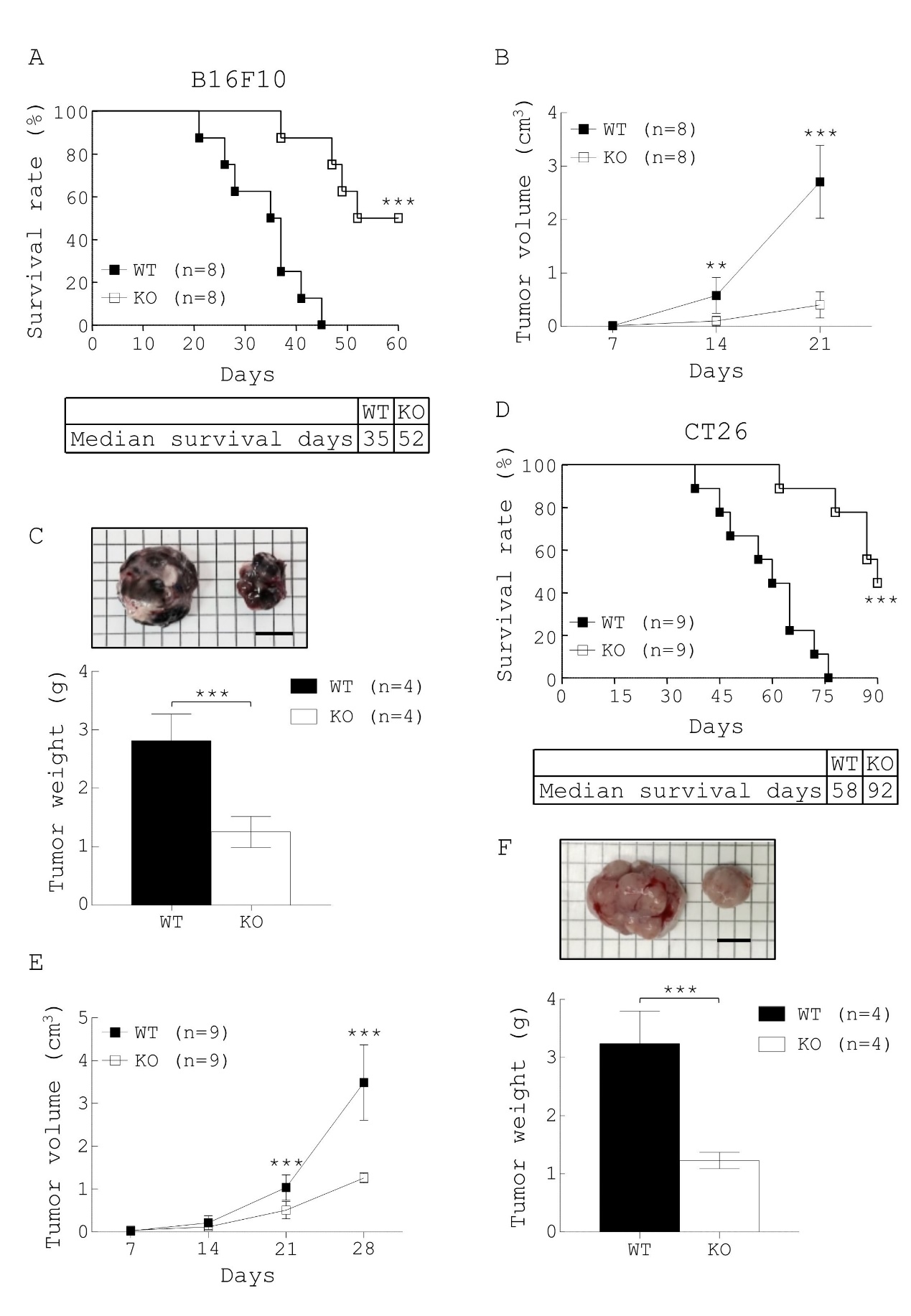
Supplementary Figures**

**Figure S1** Effect of host Pdia4 on tumor development in mice bearing melanoma and colorectal tumors. (**A - C)** GFP-expressing B16F10 cells were subcutaneously injected into WT and *Pdia4^-/-^* (KO) mice. The experimental procedure for mice was the same as Fig.2. Survival rate (**A**)**,** tumor volume (**B**), and tumor weight (**C**) of the mice were measured at the indicated days post injection. (**D - F**) GFP-expressing CT26 cells were subcutaneously injected into WT and *Pdia4^-/-^* (KO) mice. Survival rate (**D**), tumor volume (**E**), and tumor weight (**F**) of the mice were measured at the indicated days post injection. The number (n) of mice is indicated in each experiment.

**
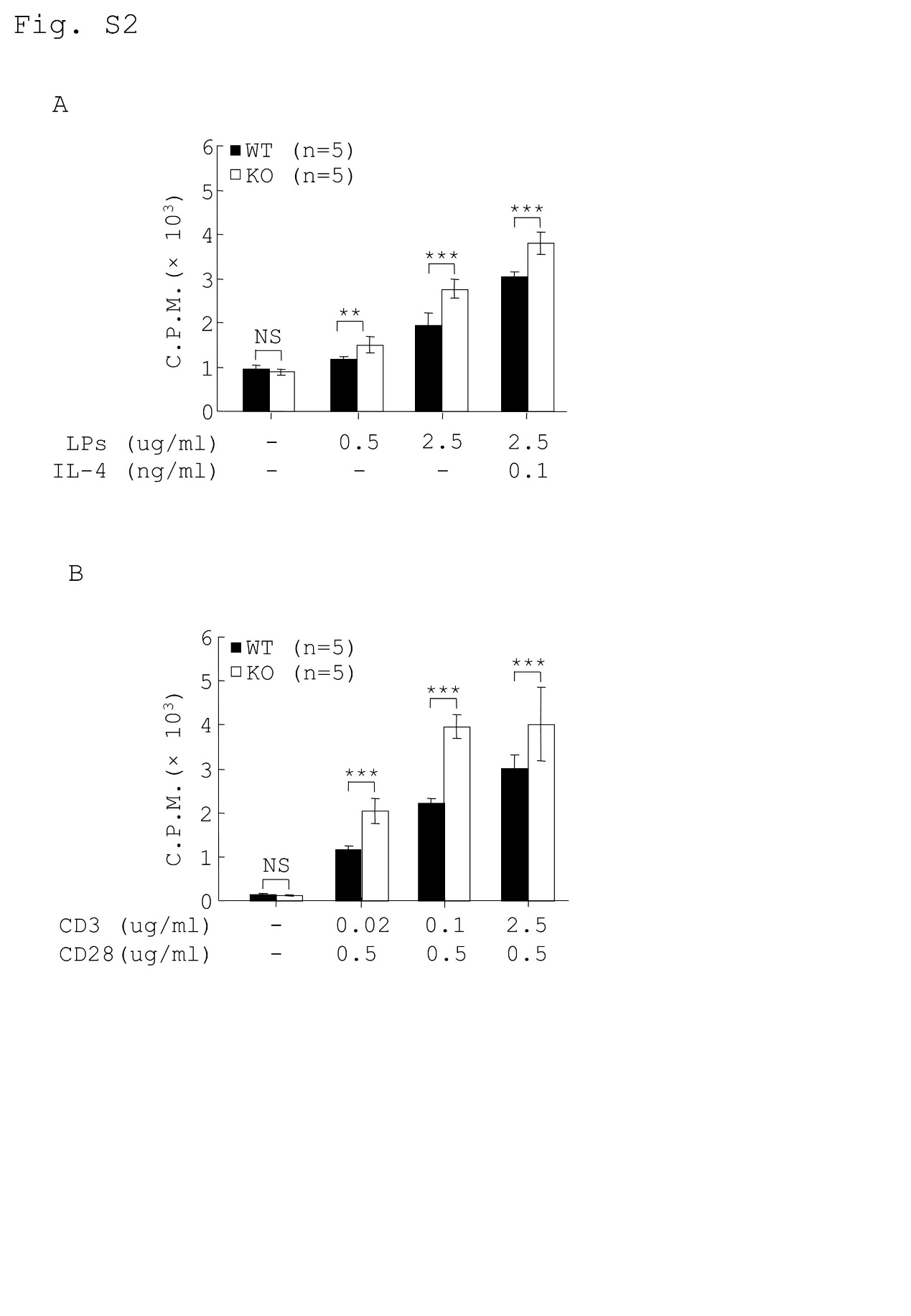
Figure S2** Effect of Pdia4 on the proliferation of stromal lymphocytes of WT and *Pdia4^-/-^* mice bearing GK1 cells. **(A**) B cells, isolated from the spleen of WT and *Pdia4^-/-^* (KO) mice bearing GK1 tumors, were stimulated using LPS and IL-4, followed by a pulse-chase procedure. Cell proliferation was measured using a β-counter. (**B**) T cells, isolated from the spleen of WT and *Pdia4^-/-^* (KO) mice bearing GK1 tumors, were stimulated using the antibodies against CD3 and CD28, followed by a pulse-case procedure. Cell proliferation was measured using a β-counter.

**
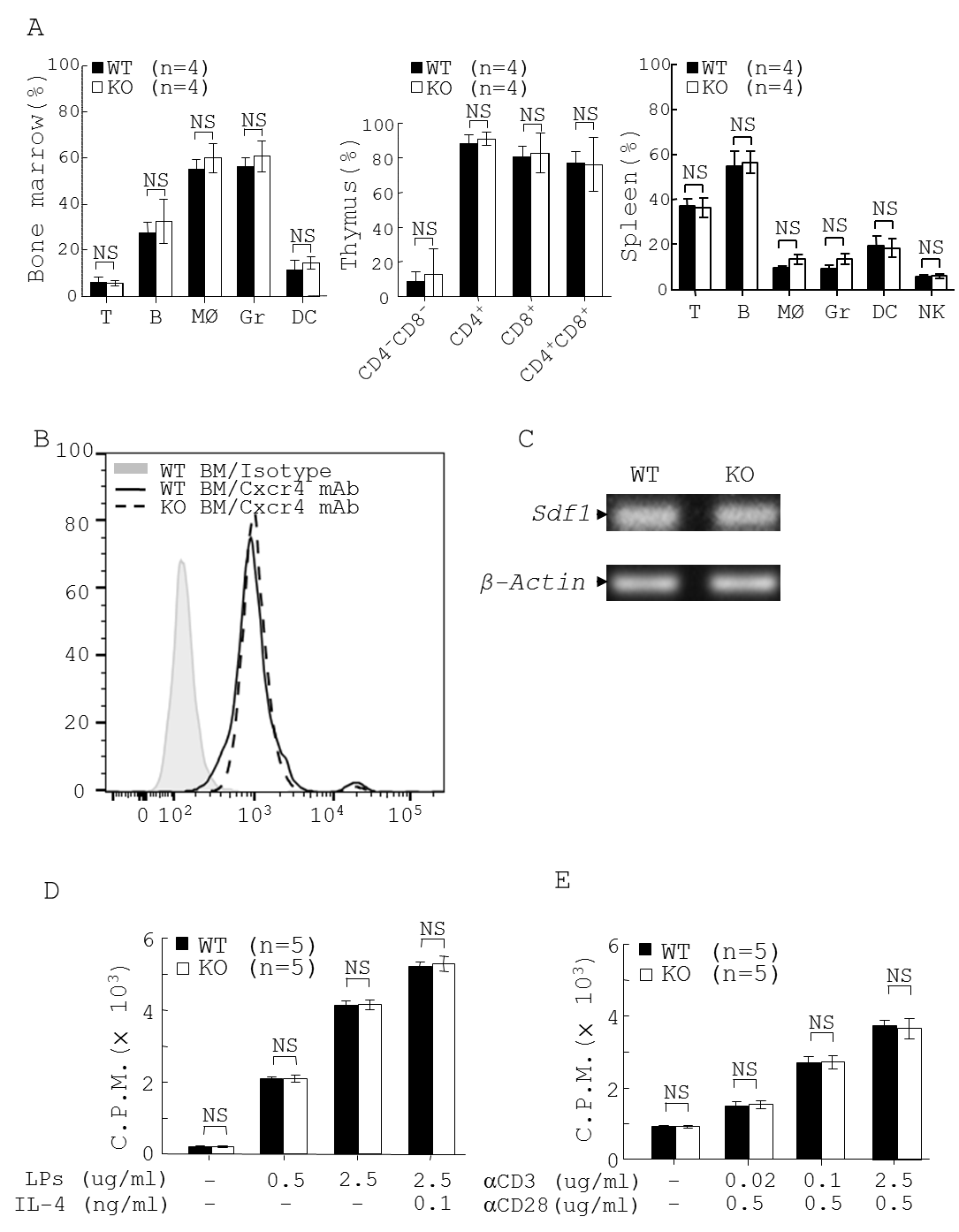
Figure S3** Pdia4 does not affect the chemotaxis of leukocytes in tumor-free WT and *Pdia4^-/-^* mice. (**A**) Cellularity analysis of bone marrows, thymi and spleens in WT and *Pdia4^-/-^* (KO) mice using a flow cytometer. (**B**) Flow cytometric analysis of bone marrow cells. The bone marrow cells of WT and *Pdia4^-/-^* (KO) mice were stained with the αCxcr4 or isotype antibody and analyzed using a flow cytometer. (**C**) PCR analysis was used to determine the expression level of *Sdf1* gene relative to β-actin gene. **(D**) B cells, isolated from the spleen of WT and *Pdia4^-/-^* (KO) mice (**A**), were stimulated using LPS and IL-4, followed by a pulse-chase procedure. Cell proliferation was measured using a β-counter. (**E**) T cells, isolated from the spleen of WT and *Pdia4^-/-^* (KO) mice (**A**), were stimulated using the antibodies against CD3 and CD28, followed by a pulse-case procedure. Cell proliferation was measured using a β-counter.

**Figure S4** Effect of host Pdia4 on the composition of stromal cells in GK1 tumor-bearing *Rag1^-/-^* and *Rag1^-/-^* *Pdia4^-/-^* mice. **(A - B**) GK1 tumor-bearing *Rag1^-/-^* (WT *Rag1^-/-^*) and *Rag1^-/-^Pdia4^-/-^* (KO *Rag1^-/-^*) mice from Fig. 4 were sacrificed. Total cells of the tumors of both mouse groups were digested with proteases and counted as described in Fig. 3A. One aliquot of the cells was stained with antibodies against stromal markers, followed by flow cytometric analysis (**A)**. The other aliquot was stained with antibodies against leukocyte markers, followed by flow cytometric analysis (**B**) as described in Fig. 3B. The percentage (left) and total cell number (right) of different cell subsets of the mouse tumors were plotted into histograms. The number (n) of mice in each experiment is indicated.

**Figure S5** Identification of the likely pathways downstream of Pdia4 in cancer stromal cells. **(A**) A flow chart identifying the likely genes implicated in the Pdia4 pathway in WT and *Pdia4^-/-^* (KO) cancer stromata on a genome-wide scale. The raw data were deposited to the NCBI Omnibus database (GSE179339). (**B**) Affymetrix array analysis of the cancer stromata of GK1 tumor-bearing WT and *Pdia4^-/-^* (KO) mice. The candidate genes from 3 replicates were selected and analyzed based on −log P (> 2.6) and z-score (> 4). (**C**) RT-PCR analysis of the genes from (**B**), Stat3, Pdia4, and Vegfa. (**D**) Immunoblotting analysis of phospho-Stat3, Stat3, Vegf family, Pdia4, and β-actin in cancer stroma from (**A)**. **(E**) Immunoblotting analysis of Akt3, Ccnd1, Pdia4, and β-actin in cancer stroma from (**A)**.

**Figure S6** Transcriptional regulation of the Vegf family by Pdia4 and Stat3 in lymphocytes. **(A**) Raji B cells were transfected with pActin-RLuc, pVegfa-FLuc and an expression vector encoding Pdia4 and/or that encoding Stat3. After 24 h, the cells were lysed and subjected to dual luciferase assays (left). The same procedure as (**A)** was conducted except that pVegfb-FLuc (middle) and pVegfc-FLuc (right) were used to replace pVegfa-FLuc, respectively. The protein level of Stat3, Pdia4 and β-actin was analyzed using immunoblotting analysis. (**B**) Jurkat T cells underwent the same procedure as (**A)**. After 16 hr, the cells were stimulated with vehicle (NS), a combination of PMA (10 ng/ml) + ION (0.5 μg/ml), Cxcl1 (1 ng/ml) and a combination of Cxcl1 (1 ng/ml) and Ccl2 (0.5 ng/ml) for an additional 8 hr. Dual luciferase assays were performed in Jurkat cells transfected with pVegfa-FLuc (left), pVegfb-FLuc (middle), and pVegfc-FLuc (right). The protein level of Stat3, Pdia4 and β-actin was analyzed using immunoblotting analysis. (**C**) Jurkat cells were transfected with pActin-RLuc, pPdia4-FLuc and an expression vector encoding Stat3 or its dominant-negative mutant, Stat3Y750F. After 24 h, the cells were lysed and subjected to dual luciferase assays. The protein level of Stat3 and β-actin was analyzed using immunoblotting analysis. (**D**) Inhibition of cell proliferation of lymphocytes by recombinant Vegf proteins. Vegfa, Vegfb, and Vegf c at the indicated dosages were incubated with splenic B (left) and T cells (right) in the presence of IL-4/LPS and αCD3/αCD28 antibodies, respectively. Cell proliferation was measured as described in Fig. 3E and 3F**.**

**FIGURE S7** Matrigel plug assays for the angiogenesis of wild-type (WT) and Pdia4^-/-^ ( KO) mice. The Matrigel plugs containing PBS and Vegfa (20 ng/plug) were subcutaneously injected into both sets of mice. After 1 week, the Matrigel plugs were removed from mice and photographed (left). After dispase digestion, their hemoglobin content was measured and re-plotted into histogram (right). The number (n) of mice is indicated in each experiment.

**Figure S8** Pdia4 RNA expression inversely correlates with prognosis of patient survival. (**A**) Survival rate and median survival time (weeks) of the patients with brain cancer (astrocytoma) whose Pdia4 expression was high (Pdia4^hi^) and low (Pdia4^lo^) was obtained from the PrognoScan RNA database (GSE4271-GLP96). (**B**) Survival rate and median survival time (weeks) of the patients with renal cell carcinoma whose Pdia4 expression was high (Pdia4^hi^) and low (Pdia4^lo^) was obtained from the PrognoScan RNA database (E-DKFZ-1). (**C**) Survival rate and median survival time (weeks) of the patients with skin cancer (melanoma) whose Pdia4 expression was high (Pdia4^hi^) and low (Pdia4^lo^) was obtained from the PrognoScan RNA database (GSE19234). (**D**) Survival rate and median survival time (weeks) of the patients with colorectal cancer whose Pdia4 expression was high (Pdia4^hi^) and low (Pdia4^lo^) was obtained from the PrognoScan RNA database (GSE12945). The number (n) of patients in each experiment is indicated.
